# Molecular Changes Concomitant with Vascular System Development in Mature Galls Induced by Root-Knot Nematodes in the Model Tree Host *Populus tremula × P. alba*

**DOI:** 10.3390/ijms21020406

**Published:** 2020-01-09

**Authors:** Fabien Baldacci-Cresp, Marc Behr, Annegret Kohler, Nelly Badalato, Kris Morreel, Geert Goeminne, Adeline Mol, Janice de Almeida Engler, Wout Boerjan, Mondher El Jaziri, Marie Baucher

**Affiliations:** 1 Laboratoire de Biotechnologie Végétale, Université libre de Bruxelles (ULB), Rue des Professeurs Jeener et Brachet 12, B-6041 Gosselies, Belgium; fabien.baldacci-cresp2@univ-lille.fr (F.B.-C.); marc.behr@ulb.ac.be (M.B.); adeline.mol@ulb.ac.be (A.M.); jaziri@ulb.ac.be (M.E.J.); 2 Unité Mixte de Recherche 1136, INRA-Université de Lorraine, Interactions Arbres-Microorganismes, Laboratoire d’Excellence ARBRE, Centre INRA-Lorraine, 54280 Champenoux, France; annegret.kohler@nancy.inra.fr; 3 CNRS, UMR 8576, UGSF—Unité de Glycobiologie Structurale et Fonctionnelle, University of Lille, F-59000 Lille, France; nelly.badalato@univ-lille.fr; 4 VIB Center for Plant Systems Biology, 9052 Ghent, Belgium; krmor@psb.vib-ugent.be (K.M.); gegoe@psb.vib-ugent.be (G.G.); wout.boerjan@psb.vib-ugent.be (W.B.); 5 Department of Plant Biotechnology and Bioinformatics, Ghent University, 9052 Ghent, Belgium; 6 VIB Metabolomics Core, 9052 Ghent, Belgium; 7 INRA, Université Côte d′Azur, CNRS, ISA, 06903 Sophia Antipolis, France; Janice.de-almeida@inra.fr

**Keywords:** cell wall, gall, metabolomics, *Meloidogyne incognita*, phloem, *Populus*, root-knot nematode, transcriptomics, xylem

## Abstract

One of the most striking features occurring in the root-knot nematode *Meloidogyne incognita* induced galls is the reorganization of the vascular tissues. During the interaction of the model tree species *Populus* and *M. incognita*, a pronounced xylem proliferation was previously described in mature galls. To better characterise changes in expression of genes possibly involved in the induction and the formation of the *de novo* developed vascular tissues occurring in poplar galls, a comparative transcript profiling of 21-day-old galls versus uninfected root of poplar was performed. Genes coding for transcription factors associated with procambium maintenance and vascular differentiation were shown to be differentially regulated, together with genes partaking in phytohormones biosynthesis and signalling. Specific signatures of transcripts associated to primary cell wall biosynthesis and remodelling, as well as secondary cell wall formation (cellulose, xylan and lignin) were revealed in the galls. Ultimately, we show that molecules derived from the monolignol and salicylic acid pathways and related to secondary cell wall deposition accumulate in mature galls.

## 1. Introduction

Phytoparasitic nematodes cause damage to plants thereby leading to substantial yield losses to annual and perennial plants worldwide [1]. Root-knot nematodes (RKN) (*Meloidogyne* spp.) are obligate sedentary parasites that enter the root of the plant host at the second juvenile stage. The larvae penetrate the root elongation zone, migrating intercellularly to the root tip and entering the vascular cylinder where they induce the trans-differentiation of parenchyma cells into multinucleate feeding cells (giant cells; GCs). Alongside, neighbouring cells divide contributing to the development of root swellings, named galls. An extensive network of xylem cells enfolding GCs is a typical anatomical feature in galls induced by RKN [2,3]. Recently, a drastic development of xylem was evidenced in poplar (*Populus tremula* × *P*. *alba*) galls 21 days after infection (dai) [4]. This profuse vascular system development most likely ensures food for the nematode development and reproduction. Upon completion of the life cycle, the adult nematode female extrudes an egg mass out of the root and once eggs hatch stage 2 juvenile nematodes, subsequent infection cycles will most likely occur in the same root [5]. 

A preliminary RT-qPCR analysis made at different time points during gall development in poplar root indicated that the expression of several genes coding for enzymes of the monolignol biosynthesis pathway was repressed, suggesting modification of the phenolic compounds profile in infected roots [4]. These genes were *HYDROXYCINNAMOYL-CoA SHIKIMATE/QUINATE HYDROXYCINNAMOYL TRANSFERASE 1* (*HCT1*) (at 7 and 14 dai), *CAFFEIC ACID 3*-O-*METHYLTRANSFERASE 2* (*COMT2*) (at 14 dai) and *FERULATE 5-HYDROXYLASE 1* and *2* (*F5H1* and *2*) (at 7, 14 and 21 dai). Monolignol pathway spans the general phenylpropanoid pathway from Phe AMMONIA LYASE (PAL) to 4-COUMARATE: CoA LIGASE (4CL) and the specific monolignol pathway from HCT to CINNAMYL ALCOHOL DEHYDROGENASE (CAD), resulting in the biosynthesis of *p*-coumaryl alcohol, coniferyl alcohol and sinapyl alcohol, which are finally polymerised into H, G and S lignin units, respectively [6].

Transcriptomic changes following *M. incognita* infection have been investigated in several species including *Arabidopsis* [7,8,9,10], tomato [11,12], soybean [13], *Medicago truncatula* [14], eggplant [15], banana [16] and bean [17]. These studies showed changes in expression of genes involved in several developmental and metabolic processes including stress response, signal transduction, phytohormones and cell wall formation, as well as phenylpropanoid biosynthesis.

Several genes associated with procambium proliferation and vascular differentiation were differentially regulated on 5 and 7 dai RKN-induced galls in *Arabidopsis* [10]. Herein, in order to better document at the molecular level this intense vascular development in RKN-induced galls in a woody species, a transcriptomic analysis of 21 dai galls compared to uninfected root was performed. We focused on the induction of the transcriptional programme related to vascular system differentiation and maturation as well as to regulation of phytohormones biosynthesis and signalling. We further describe the expression pattern of genes involved in primary and secondary cell walls formation and modification. As well, a targeted metabolomic analysis was performed to highlight the changes occurring in the pool of soluble phenolics related to monolignol metabolism in galls.

## 2. Results and Discussion

### 2.1. Transcriptome Overview of Galls at Late Stage

A RNA-Seq analysis was performed on 21 dai galls and corresponding uninfected poplar roots. A total of 70.4–80.8% of the reads were successfully mapped to the substituted genome sequence of *P. tremula* × *P. alba* 717-1B4 (Table S1). A total of 8043 genes present a differential transcript abundance (taking into account transcripts with a fold change >2, minimum 20 reads and adjusted *p*-value < 0.05; Table S2). Among these genes, 4549 were up- and 3494 down-regulated in galls compared to uninfected roots.

**Figure 1:**
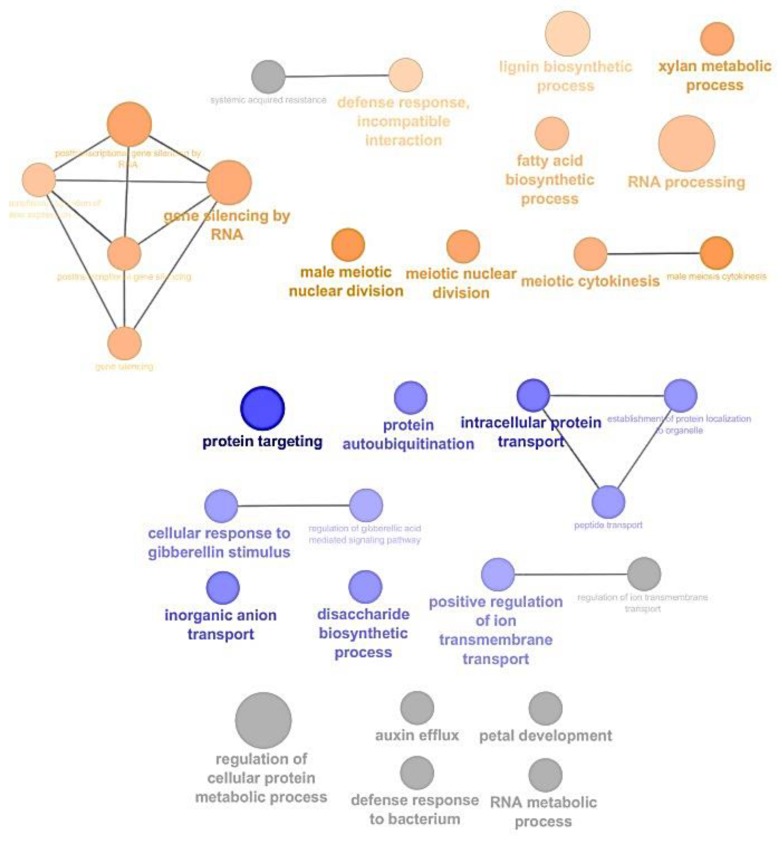
Gene ontology (GO) enrichment performed with the subset of genes showing –2 > fold change > 2 in Cytoscape using ClueGO and CluePedia. Biological processes with genes overrepresented in 21 dai galls and uninfected roots are shown in orange and blue, respectively. All GO are listed in Table S3.

All the differentially expressed genes were represented in ClueGO and CluePEDIA [18,19] within Cytoscape using their *Arabidopsis thaliana* homologs ID (Table S3 and Figure 1). As shown in Figure 1, gene ontologies (GO) such as “xylan metabolic process”, “lignin biosynthetic process”, “male meiotic nuclear division” and “gene silencing by RNAi” were prevalent in galls. On the other hand, “protein targeting”, “intracellular protein transport”, “inorganic anion transport” and “cellular response to gibberellin stimulus” were associated with genes downregulated in galls compared to roots. In the light of these GO, the next paragraphs are devoted to the study of genes involved in vascular tissue development, primary and secondary cell wall formation and modification, and phytohormones biosynthesis and signalling.

### 2.2. Gall Transcriptome Reveals Changes in Gene Expression Associated to Primary Cell Wall Formation and Modification

The establishment of a nematode feeding site steers the development of 4–10 GCs that have a peculiar cell wall pattern, such as accumulation of cellulose and mixed-linked glucans (MLG) [4]. The nematode nutrient acquisition through GC relies on the formation of *de novo* formed vascular tissue. Primary and secondary cell walls (PCW and SCW) are deposited in both GC and vascular tissues [20]. Our transcriptomic dataset highlighted major changes in the expression of genes from the *CesA* and *CesA-like* (*CSL*) families (Table 1). For instance, the homologs of *Arabidopsis CesA3*, *CesA6* and *CesA9* were upregulated in the galls, together with several *CSL* of multiple families (A, C, D, E, G). In *Arabidopsis* roots infected with *M. incognita*, *CesA* expression peaked 5 days post-inoculation, with PCW *CesA* being preferentially localized to cells neighbouring GC [20], i.e., young vascular cells. Our data may therefore indicate that cell formation and/or elongation, which both require cellulose biosynthesis for PCW formation, were ongoing in 21 dai galls.

CSL are part of the cellular machinery producing several cell wall matrix polysaccharides, including xyloglucan (CSLC), mannan (CSLA), β-1,4-glucan and MLG [21]. Genes coding for enzymes involved in cell wall modification were also differentially regulated in the galls. Among these, two homologs of *Arabidopsis XYLOGLUCAN ENDOTRANSGLUCOSYLASE/HYDROLASE* (*XTH*) 9 and one homolog of *AtXTH16* (Potri.002G236200) were strongly upregulated in galls (log_2_ FC of 2.34, 3.03 and 3.78, respectively). XTHs cleave and ligate xyloglucan chains [22]. *AtXTH9* is expressed in shoot apices and elongating cell tissues [23], thus their poplar homologs may contribute to the cell wall rearrangement occurring in extending xylem cells. The poplar *ARBORKNOX1* (*ARK1*) regulates biological processes related to cambial activity and xylem differentiation during secondary growth and its overexpression results in the upregulation of Potri.002G236200 [24]. The extensibility of PCW is controlled in specific regions of the extracellular matrix, the so-called “biomechanical hotspots”, where expansins, which are essentially upregulated in the galls (Table S2), loosen the cellulose-xyloglucan interaction [25].

### 2.3. Structural Genes of Lignification and SCW Formation are Induced in Galls

Among SCW structural genes upregulated in galls (Table S2), we found genes involved in monolignol biosynthesis such as *PAL* (Potri.016G091100, Potri.006G126800), *CINNAMATE 4-HYDROXYLASE* (*C4H*) (Potri.018G146100), *CINNAMOYL-CoA REDUCTASE* (*CCR*) (Potri.001G045500), *CAFFEOYL-CoA O-METHYLTRANSFERASE* (*CCoAOMT*) (Potri.008G136600, Potri.009G099800) and *CAD* (Potri.016G078300) (Table 2). Fifteen laccases-encoding genes homologous to either *AtLAC4*, *AtLAC11*, *AtLAC12,* or *AtLAC17* (involved in lignin polymerisation; [26]) were upregulated in galls (Table S2), together with 3 genes (Potri.016G132700, Potri.013G083600 and Potri.014G143200) homologous to *AtPRX52*, coding for a class III peroxidase involved in lignin polymerisation [27]. A gene annotated as *ALCOHOL DEHYDROGENASE 1* (Potri.002G072100) was among the most highly expressed in our dataset, with average normalised counts of 2369 in galls vs. 309 in roots. The corresponding protein of this gene accumulates during stem development [28] with a maximum observed in internode with lignified wood and phloem tissues, and may therefore be involved in monolignol biosynthesis. Other genes involved in monolignol biosynthesis were downregulated in galls such as several *4CL*, *CAD* and *HCT* members as well as *F5H1* and *F5H2*, required for the biosynthesis of sinapaldehyde and sinapyl alcohol [6] (Table 2). Lignans biosynthesis may also be differentially regulated in the galls. Three genes annotated as *PHENYLCOUMARAN BENZYLIC ETHER REDUCTASE* (*PtrPCBER1*/Potri.002G034400, *PtrPCBERp5*/Potri.001G133200 and *PtrPCBERp8*/Potri.003G100200), based on their homology with *AtPCBER* [29], were more expressed, while one was downregulated in galls (*PtrPCBERp4*/Potri.001G133300). These genes are also similar to *PINORESINOL-LARICIRESINOL REDUCTASE*, another gene of the lignan biosynthetic pathway [30]. More expressed in the galls were two genes of C1 metabolism linked to monolignol methylation, homologs of *Arabidopsis S-ADENOSYLMETHIONINE SYNTHETASE* 3 (Potri.013G004100) and *METHYLENETETRAHYDROFOLATE REDUCTASE 2* (Potri.007G147300) [31].

Homologs to genes involved in xylan reducing-end sequence formation (*PARVUS*), backbone elongation (*IRX9*/*PtrGT43A*, *IRX10*) and backbone substitution (*ESK1*, *GXMT1*, *GXM3*, *GUX1*, *RWA2*, *RWA3* and *TBL35*) were upregulated in the gall together with three homologs of *IRX15-L* (Table S2), which is required for xylan biosynthesis [32]. More expressed in galls were genes coding for PtCesA8-A (Potri.011G069600), a cellulose synthase partaking in cellulose deposition in SCW [33] and poplar homologs of *AtTED6* (Potri.002G072000) and *AtTED7* (Potri.007G108100, Potri.005G060700). TED6 and TED7 are candidates to take part of the SCW CesA complex during xylem formation [34]. The same trend was observed for the expression of *XCP1* and *XCP2*, which are VND6-VND7-regulated during programmed cell death in xylem tissue [35,36]. We may also suppose that PtCesA8-A may contribute to cellulose deposition in GCs to cope with the cellular turgor pressure [20]. Also upregulated in galls were several extensins (Potri.002G070100 and Potri.005G190100) which can covalently crosslink along the cell wall. As these proteins cause recalcitrance biomass in poplar [37], it is plausible that they are present in, and interact with, SCW of GCs and xylem and their building blocks xylan and lignin in xylem [38,39].

Altogether, our data strongly suggest that galls induced by RKN undergo a SCW programme within the xylem tissue resulting from the infection. So far, most of the articles studying RKN interaction highlighted the differential regulation of genes of the phenylpropanoid pathway [7,12,13,40] but generally with a focus on GC development in herbaceous species (*Arabidopsis*, tomato, soybean). Our data indicate that this transcriptional regulation of monolignol biosynthesis is part of the gene network involved in the xylem development adjacent to GCs, together with xylan biosynthesis and cellulose deposition.

### 2.4. Galls are Associated with Major Expression Changes of Transcription Factors Regulating Xylem and Phloem Development and Cell Wall Formation

Galls are characterised by the development of *de novo* formed xylem cells, which are considered to be similar to wound-type xylem elements, and by the formation of phloem sieve elements [2]. As shown in Table 3, transcription factors (TFs) and other genes involved in vasculature patterning and xylem differentiation were found to be upregulated in the 21-day-old galls as compared to uninfected roots. This included the homolog of *Arabidopsis* HD-ZIP III *AtHB8* (Potri.006G237500), which promotes vascular cell differentiation to xylem [41,42]. As well, *LBD1* (Potri.010G217700), which is involved in secondary phloem and ray cell formation [43], *ALTERED PHLOEM DEVELOPMENT* (*APL*), a phloem precursor [44], and *KANADI 1* and *KANADI 2*, coding for positive regulators of phloem formation through regulation of auxin flow [42,44], were upregulated. Two genes homologous to *ACAULIS 5* (*ACL5*), a direct target of AtHB8 with a role during xylem vessels and fibres specification, were upregulated in the galls. More specifically, *ACL5* prevents premature xylem cell death to allow their complete elongation and secondary cell wall deposition [35,45]. Two genes closely related to *AtPIN1*, involved in auxin efflux during early stages of procambial cell differentiation under the control of AtHB8 [35], as well as Potri.001G049700 and Potri.012G019400, coding respectively for PtCLE2 and PtCLE38 (homologous to *Arabidopsis* CLE41-CLE44/TDIF) peptides, displayed an expression pattern similar to *ACL5* homologues. The overexpression of *PtCLE38* and its cognate receptor kinase *PXY*, driven by phloem-specific and cambium-specific promoter, respectively, dramatically increases xylem cell density in hybrid aspen [46]. Overall, this transcriptional landscape indicates an ongoing formation of phloem and xylem tissues in the galls. These results are complementary of those gained from a transcriptomic time-course analysis of galls induced by RKN in *Arabidopsis* [10]. The authors report at 5 and 7 dai an upregulation of genes associated with procambium, such as *AtHB8* and *PXY*, while xylem-associated genes such as *XCP*s and *TED*s were slightly downregulated. In addition, phloem markers (*APL*, *SUC2*) were less upregulated than procambium genes. It is therefore consistent that genes associated with xylem and phloem differentiation are upregulated at later stages of gall development, i.e., 21 dai, to drive the maturation of the vascular system required for nematode feeding. For instance, 16 genes homologous to *SIEVE-ELEMENT-OCCLUSION-RELATED 1* (AT3G01680), which is a marker of phloem development [10], were strongly upregulated in the galls (Table S2). Our data indicate that the gall maintain a pool of procambial cells late during its development, possibly through PtCLE2 or the secretion of a CLE-like peptide by the nematode [2], to support the formation of vascular tissue required for the completion of nematode life cycle.

Xylem cells undergo a SCW deposition program, including the biosynthesis of the main polysaccharide xylan and lignin. This SCW network is transcriptionally regulated by NACs and MYBs at three hierarchical levels [47,48,49,50]. The master regulator *PtrSND1-B1* (Potri.014G104800) and several downstream TFs, such as the second level *PtrMYB074* (Potri.015G082700), *PtrSND2/3-A1* (Potri.011G058400), *PtrSND2/3-B1* (Potri.007G135300), as well as *PtrVND7*-1 (Potri.019G083600) and *PtrVND7-2* (Potri.013G113100) were more expressed in galls, thereby confirming the expression pattern of genes involved in SCW formation. Also upregulated were 2 genes homologous to *WEE1 KINASE HOMOLOG* (Potri.T034700 and Potri.014G132400, Table S2), which arrests cell cycle at the G1/S step in response to DNA damage [51], providing a checkpoint mechanism before cellular division. WEE1 prevents premature tracheary element differentiation upon DNA damage to maintain meristem identity through a VND7-regulated mechanism [52]. WEE1 is also a crucial element during gall development, as an *Arabidopsis wee1* knockout line shows repressed infection and reproduction of RKN [53], suggesting that plant DNA integrity is an important element of the life cycle of the nematode. The hyperactivation of cell division occurring in galls is likely to provoke DNA damage [53]. We may speculate that WEE1 is involved in the maintenance of the pool of procambial cells, by preventing vascular differentiation through detection of DNA damages induced by nematodes.

However, some of the TFs controlling SCW formation were downregulated in galls, such as *PtrMYB088* (Potri.018G095900) and *PtrMYB175* (Potri.017G017600) which are regulated by PtrMYB021, PtrMYB074, or both [47]. PtrSND1-B1 directly regulate the expression of *PtrMYB021* and *PtrMYB074*, which are transcriptional activators of fibres and vessels in poplar [47]. *KNOX* homologous to *Arabidopsis BLH4* (*PtrWBLH2*/Potri.005G129500), *BLH6* (*PtrWBHLH3*/Potri.004G159300, Potri.009G120800), *BLH7* (*PtrWBLH1*/Potri.002G031000) and *KNAT3* (Potri.018G114100 and Potri.006G259400) were also repressed in galls (Table 3). Heterodimers between BLH and KNAT are able to repress the expression of monolignol biosynthetic genes [54], while BLH6 is able to repress SCW formation [55]. Another TF from the KNOX family, KNAT2/6b, inhibits the differentiation of xylem cells through repression of several *NAC* and *MYB* TFs [56]. The development of the gall vascular system may therefore be associated with a repression of genes preventing the differentiation of these specialised cells, possibly guaranteeing sugar and water sources throughout the nematode life cycle. Since these genes are, during normal development, co-expressed with the positive regulators of this biological process [50,55,56], we may hypothesise that their down-regulation in the galls is tuned by an exogenous factor, as for example a compound secreted by the nematode [57]. Finally, one can hypothesise that the down-regulation of these repressors leads to drastic xylem development to support the completion of the nematode life cycle. Alternatively, expression of these genes may be higher in GCs (to prevent further differentiation) than in xylem cells; assuming that gene transcription is significantly enhanced in GCs as compared to xylem, the resulting overall balance shows gene repression in galls.

The expression of the *Arabidopsis* homologs of *XND1* and *VNI2*, which inhibit xylem differentiation and lignocellulose biosynthesis [58,59] and xylem vessel formation [60], respectively, were downregulated in galls. Potri.013G109300, homologous to the MYB R2-R3 subgroup *AtMYB3*, as well as Potri.017G017600, Potri.002G073500 and Potri.005G186400 (homologous to *AtMYB52*) were also downregulated in galls. AtMYB3 and AtMYB52 negatively regulate the lignification process [61,62]. These data therefore suggest that the de-repression of the SCW/lignin transcriptional network and of xylem differentiation is part of the nematode-induced gall development program.

### 2.5. Expression of Genes Related to Phytohormones is Modified in Galls

Nematodes alter the metabolism of the host to favour cell division, leading to gall formation. The GO analysis highlighted the up-regulation of genes involved in nuclear division and cytokinesis (Figure 1). These genes included *FUSED* (*FU*) and *MINICHROMOSOME MAINTENANCE 3, 4, 5, 6, 7, 8* and *10* (*MCM*3, 4, 5, 6, 7, 8, 10), which are important actors of cell division [63,64] (Table 4), and genes encoding cyclins (Table S2), which activate the cell cycle in infected roots, as similarly observed in RKN-induced galls in *Arabidopsis* [65]. The increased cell division in galls is (partially) activated through cytokinin signalling [57]. Accordingly, several homologs of the *ARABIDOPSIS RESPONSE REGULATOR* (*ARR*) genes, which are the final acceptors of the cytokinin signalling pathway, were upregulated in the galls (*ARR2*, *9*), while expression of *ARR12* was repressed. ARR2 and ARR12 are type-B regulators (mediating cytokinin-dependant transcriptional activation) while ARR9 is a type-A regulator (negative regulator of cytokinin signalling; [66]). The expression of *ARR9* in galls is much higher than the 2 type-B, suggesting that cytokinin signalling is repressed. Cytokinins in association with auxins are determinant factors of phloem formation in galls [2]. Previous works noted the upregulation of another type-A, *ARR5*, in the cells surrounding the GC, i.e., phloem [67], while no response related to type-B ARR was observed in *Arabidopsis* galls [68]. Our data also indicate that positive cytokinin-dependant gene expression is repressed in 21 dai galls. It has been suggested that this gene expression pattern may prevent the differentiation of protophloem, which is devoid of companion cells as observed in RKN-induced galls, into metaphloem [68].

Auxin is another key hormone for gall development, with roles ranging from cell cycle activation to cell wall remodelling [2,69]. Our data do not point to a prominent role of auxin in 21 dai galls. First, the expression of *YUCCA* genes, which encode the rate-limiting step enzyme in auxin biosynthesis, were downregulated in galls. Second, the large majority of the *AUXIN RESPONSE FACTORS* (such as homologs of *ARF2*, *ARF10* and *ARF13*) and of the *SMALL AUXIN-UP RNA* genes (*SAUR*) were consistently repressed in galls (Table S2). The expression of several genes homologous to *PIN*, which pump the auxin out of the cell, was significantly upregulated in galls (Table 4), where they may contribute to auxin delivery in GCs [69]. We may speculate that the sampled material (21 dai) has developed to a stage where auxin signalling is decreased. Indeed, an auxin response has been detected before the differentiation of phloem cells [68], at earlier stages of gall development (between 1 and 2 weeks after infection). The upregulation of several phloem marker genes in our galls suggest that weak auxin signalling is a consistent trait of vascular differentiation in RKN-induced galls.

Several genes involved in ethylene biosynthesis and signalling showed altered expression patterns in galls (Table S2). Indeed, two out of three genes homologous to the *ETHYLENE-FORMING ENZYME ACO4* were upregulated in galls, together with several TFs activating downstream ethylene response, the *ETHYLENE RESPONSIVE ELEMENT BINDING FACTORS* (*EREBF*, including *ERF1*; [70]). Ethylene signalling pathway enhances lignin biosynthesis, which contribute to the strengthening of xylem SCWs [71]. Ethylene may thus be an important player of xylem gall development.

Our data did not show a clear trend regarding the potential involvement of gibberellic acid (GA) in galls (Table 4), probably because of the gall developmental stage (21 dai) analysed here. Indeed, the expression of two genes encoding enzymes catalysing, respectively, the GA biosynthesis rate limiting step, *GA20OX2* (*GIBBERELLIN 20 OXIDASE 2*), and the last reaction of this pathway, *GA3OX1*, were strongly downregulated in galls (log_2_ FC of −1.80 and −4.20 for *GA20OX2*; −2.28 and −2.82 for *GA3OX1*). In addition, *GA2OX4*, a gene responsible for GA inactivation [72] was also downregulated in galls. However, three GA-induced genes (2 homologs of *GASA14* and one homolog of *GASA6*) were upregulated in galls and genes coding for the transcriptional regulators repressing GA response, the DELLA proteins GAI and RGA [73], were strongly downregulated (Table 4). Despite the down-regulation of the biosynthetic genes, it seems therefore that galls are characterised by an increased response to GA. It is plausible that GA are synthetized in the roots [74] and activate the signalling pathway in the galls. This phytohormone is required for pectin biosynthesis and homogalacturonan esterification and is involved in the modification of the cellulose-xyloglucan network [75]. GA also upregulate the expression of the three SCW *CesA*s in *Arabidopsis* [76]. This gene expression pattern suggests that GA are an important factor in the RKN-plant interaction both in young cells remodelling their PCW and in cells undergoing a SCW formation.

Finally, salicylic acid (SA) is a phytohormone of critical importance during plant response to various biotic stresses. The SA biosynthesis gene *ICS1*, encoding ISOCHORISMATE SYNTHASE 1, was found to be slightly (log_2_ FC 1.16) upregulated in galls as compared to uninfected roots. A higher expression of four genes homologous to *PATHOGENESIS-RELATED 1* (*PR1*), a marker of SA-related plant defence was also observed. Furthermore, high expression of a gene homologous to *WRKY53*, a SA-inducible TF [77] was observed in galls (Table 4). SA strongly antagonizes the jasmonic acid (JA) signalling pathway [78] and down-regulates the expression of several genes of the JA biosynthetic pathway [79]. Accordingly, the gene coding for JASMONATE RESISTANT 1 (JAR1) was repressed in galls and those encoding JASMONATE-ZIM-DOMAIN PROTEIN (JAZ) 5, 6 and 10 (Table S2) were upregulated. JAR1 catalyses the formation of the bioactive jasmonyl-isoleucine (JA-Ile) conjugate, while JAZ proteins repress the JA signalling pathway [80]. JA favours secondary growth and more specifically the formation of phloem fibres in herbaceous species [81,82]. Since phloem developing in galls retains a protophloem identity [68], we may hypothesise that phloem fibre formation, a strong differentiation process, is repressed in galls. The expression pattern of genes related to JA biosynthesis and signalling may be linked to the absence of phloem fibres in galls. Consequently, our data suggest that JA is not a key phytohormone partaking in vascular patterning of galls.

Overall, as already reported for other plant-RKN interactions [83], many genes coding for hormone metabolism and signalling are differentially expressed in 21 day-old poplar galls. Although the role of these different hormones in the plant-RKN interaction is not clearly elucidated, these are obviously involved in gall formation, development and metabolism. It is therefore likely that gall development is closely associated with moving transcriptional signatures of phytohormones biosynthesis and signalling, as already shown in galls from 2 to 14 dai [20].

### 2.6. Comparative Metabolic Profiling of Mature Gall and Root in Poplar

To strengthen the results obtained by RNA-Seq with potential alterations in the soluble phenolic pool, a phenylpropanoid-targeted metabolomic analysis of methanolic extracts from uninfected roots and 21 dai galls was performed. A total of 21045 mass-to-charge ratio (*m/z*) features, including ones associated with compounds from both plant and nematode origin, were identified. To gain insight into the metabolites that differ between the organs, a one-way ANOVA was performed here (*p*-value ≤ 0.05). Following this analysis, 12921 features differed in abundance between uninfected roots and galls (Table S4). As shown in Table 5, among the principal identified *m/z* features more abundant in galls, phenylpropanoids derived from hydroxycinnamic acids such as ferulic and caffeic acids were prevalent. Four guaiacyl/syringil trilignols were slightly more abundant in galls, suggesting a higher production of monolignols and lignin/lignans.

Tryptophan and SA-derived molecules including tremulacin, salicyloyl salicin, salicortin and salireposide, also showed an increased abundance in galls. Tryptophan accumulation may result from, (i) the increased activity of the shikimate pathway that supplies phenylalanine for phenylpropanoids biosynthesis [84], and (ii) the biosynthesis of indole-conjugates as defence metabolites, requiring tryptophan as substrate [85]. Salicortin, salireposide and tremulacin are detected in higher amount in the phenolic fraction of a transgenic poplar stem with altered *LACCASE 3* expression, as compared to the wild type [86]. This transgenic line has no lignin phenotype but show defects in the structure of cell wall of the xylem fibres and it has been suggested that such phenolic compounds may cross-link with cell wall components, especially in PCW and middle lamella. Overexpression of *EgMYB88* induces salicortin, salireposide and tremulacin accumulation in the stem of poplar [87]. EgMYB88 may control the biosynthesis of phenylpropanoids (monolignols, flavonoids and phenolic glycosides) in the cambium and in young differentiating xylem cells of woody species [87]. It is also relevant that Potri.013G074500, encoding a benzoyl-*CoA*:salicylalcohol *O*-benzoyltransferase involved in salicinoids biosynthesis [87], was upregulated in galls (Table S2). The accumulation of phenolic glycosides in the altered *LACCASE 3* and *EgMYB88* backgrounds, likely indicate that they play a role in SCW formation in galls. From a plant-nematode interaction perspective, tremulacin, salicyloyl salicin, salicortin and salireposide show anti-herbivore properties [88]. Moreover, SA has a strong nematicidal activity [89], supporting the hypothesis that these four molecules are part of the plant response to nematode infection. For instance, salireposide consists in the conjugation of salicin with 1-hydroxy-6-oxo-2-cyclohexene-1-carboxylic acid (HCC), the precursor of 6-hydroxy-2-cyclohexen-on-oyl (HCH), which is toxic to herbivores [90].

The six most down-accumulated identified compounds (<0.1× in galls are a ferulic acid derivate, chlorogenic acid (CGA), as well as 3 *p*-coumaric acid and 1 caffeic acid derivates. CGA is involved in root hair formation [91], lignin biosynthesis [92], and in plant defence [85,93]. CGA competes with SA for the pool of *p*-coumarate, a molecule required for the biosynthesis of both compounds [94,95]. The pool of CGA may therefore decrease because the biosynthesis of SA derivates and monolignols are favoured in galls.

## 3. Materials and Methods

### 3.1. Plant and RKN Material, Growth Conditions and Nematode Infection

Poplar (*P. tremula* × *P. alba* clone INRA 717-1B4) was grown aseptically as described in [96]. RKN infections were as described in [4].

### 3.2. RNA-Seq

Total RNA was extracted from 21 dai galls and corresponding uninfected poplar roots (*n* = 3) using the Plant RNA Isolation Kit (Agilent, Santa Clara, CA, USA) with a 15 min incubation step and a 5 min centrifugation step (20,000× *g*) added before prefilter column application. RNA quality controls were performed using a BioAnalyzer RNA Nano (Agilent, Santa Clara, CA, USA). All samples presented a RIN comprised between 9 and 10. TruSeq mRNA Stranded libraries were performed following Illumina guidelines.

Library control quality and library quantifications were performed using the QIAxcel DNA (QIAgen, Hilden, Germany) and the Kapa Library Quantification (Kapa Biosystems, Amsterdam, Netherlands) kits, respectively. After pooling and normalization of libraries, sequencing was done on a NextSeq500 Sequencing System (Illumina, San Diego, CA, USA). RNA quality test and sequencing were completed by the GIGA genomic platform (Liege University, Liege, Belgium).

Raw reads were trimmed for quality and mapped to the substituted genome sequence of *P. tremula* × *P. alba* 717-1B4 (http://aspendb.uga.edu/index.php/databases/spta-717-genome; [97,98]) using CLC Genomics Workbench v9.5.2 and the primary transcripts only. For mapping, the minimum length fraction was 0.9, the minimum similarity fraction 0.8, the maximum number of hits for a read was set to 1, mapping was not strand specific and only intact paired reads were counted (Table S1). To identify differentially regulated *Populus* transcripts, the Linear Models for Microarray and RNA-Seq Data (limma) package [99] in R (R Development Core Team, 2013, Vienna, Austria) and the raw counts from the CLC mapping were used as described in [100]. The complete RNA-Seq data was submitted to GEO (GSE112673).

### 3.3. Validation of the RNA-Seq Results

The same RNA was used for RNA-Seq and RT-qPCR analyses. RT using ProtoScript^®^ II First Strand cDNA Synthesis Kit (NEB, Ipswich, MA, USA) was made to perform validation on gall vs. root (500 ng RNA per RT) and diluted 20×. Next, qPCR (*n* = 2) was achieved using *PT1* (Potri.002G127700.1), *CYC063* (Potri.005G240200.1) and *UBP22* (Potri.018G017000.1) as reference genes [4]. The RNA-Seq results were validated for 7 genes using RT-qPCR. The observed fold changes were consistent between the two methods (Table S5).

### 3.4. Metabolomics

Uninfected roots and 21 dai galls (*n* = 4) were frozen in liquid N_2_ and grinded. A total of 100 mg of gall and 200 mg of root samples were extracted with 2 volumes of methanol. After agitation of 30 min at room temperature and centrifugation, supernatants were dried for 2 h in a speedvac and dissolved in 100 µl of cyclohexane and 100 µl of milliQ water. After centrifugation, 3 µl water phases were analysed by reversed phase Ultrahigh Performance Liquid Chromatography (Acquity UPLC I-Class system; Waters, Milford, MA, USA) coupled to a Quadrupole-Time-of-Flight Mass Spectrometer (Vion IMS QTOF; Waters) via an Electrospray Ionization source operated in the negative ionization mode. Chromatographic and MS conditions were as previously described [101], chromatogram processing was done with Progenesis QI v2.3 (Waters). Using an in-house database, identifications (built with Instant Jchem for Excel, ChemAxon, Budapest, Hungary) were based on the retention time, precursor *m/z* value and MS spectra recorded in data-independent acquisition mode (low energy was set at 6 eV, high energy was ramped from 10 to 40 eV). LC-MS data were normalised for the sample dry weight and further statistically analysed using R v. 3.4.3. Missing data were replaced by a random number drawn from a uniform distribution between 1 and 5 [sample() function].

The homogeneity of variance between galls and uninfected roots was checked using the bartlett.test() function and a Box-Cox transformation was applied whenever necessary. A one-way Analysis of Variance (ANOVA) model was computed with the lm() function in which weighing was necessary due to the loss of one root sample.

## 4. Conclusions

Galls in poplar result from the proliferation of xylem and phloem tissues to support the feeding of the nematode through the GCs. These biological processes rely on the preferential expression of several TFs and downstream biosynthetic genes, notably those involved in SCW formation. Nematode is therefore able to manipulate plant transcriptional regulation to mimic a developmental differentiation process favouring its own life cycle, at the expense of plant metabolism. However, the identification of the nematode molecular signals steering this programme will require further investigations. It seems that phytohormones such as cytokinins, GA and SA shape the development of galls. The targeted analysis of metabolites related to monolignols and salicylated products confirmed the reaction of the plant after nematode infection, which consists in the biosynthesis of monolignol intermediates and phenolic glycosides as lignin building blocks for xylem SCW.

## Figures and Tables

**Table 1:** Differentially expressed genes involved in PCW formation and modification in 21 dai galls versus uninfected roots.

Poplar ID	log_2_ FC	*Arabidopsis* ID	*Arabidopsis* Gene Name
Potri.005G087500	1.33	AT5G64740.1	*CESA6*, *E112*, *IXR2*, *PRC1*
Potri.005G194200	1.06	AT2G21770.1	*CESA09*, *CESA9*
Potri.016G054900	1.49	AT5G05170.1	*ATCESA3*, *ATH-B*, *CESA3*, *CEV1*, *IXR1*
Potri.006G052600	1.64	AT5G05170.1	*ATCESA3*, *ATH-B*, *CESA3*, *CEV1*, *IXR1*
Potri.006G004200	2.45	AT1G55850.1	*ATCSLE1*, *CSLE1*
Potri.001G369100	−1.12	AT1G55850.1	*ATCSLE1*, *CSLE1*
Potri.003G142400	2.04	AT4G23990.1	*ATCSLG3*, *CSLG3*
Potri.003G142300	3.43	AT4G23990.1	*ATCSLG3*, *CSLG3*
Potri.003G142500	2.29	AT4G23990.1	*ATCSLG3*, *CSLG3*
Potri.009G149700	1.46	AT5G22740.1	*ATCSLA02*, *ATCSLA2*, *CSLA02*, *CSLA2*
Potri.004G189000	2.12	AT5G22740.1	*ATCSLA02*, *ATCSLA2*, *CSLA02*, *CSLA2*
Potri.013G082200	1.12	AT3G03050.1	*ATCSLD3*, *CSLD3*, *KJK*
Potri.014G125100	1.72	AT1G02730.1	*ATCSLD5*, *CSLD5*, *SOS6*
Potri.002G200300	1.86	AT1G02730.1	*ATCSLD5*, *CSLD5*, *SOS6*
Potri.005G146900	1.31	AT4G07960.1	*ATCSLC12*, *CSLC12*
Potri.002G114200	1.45	AT4G07960.1	*ATCSLC12*, *CSLC12*
Potri.018G009300	1.17	AT4G31590.1	*ATCSLC05*, *ATCSLC5*, *CSLC05*, *CSLC5*
Potri.006G270900	1.43	AT4G31590.1	*ATCSLC05*, *ATCSLC5*, *CSLC05*, *CSLC5*
Potri.002G236200	3.78	AT3G23730.1	*XTH16*
Potri.019G125000	2.34	AT4G03210.1	*XTH9*
Potri.013G152400	3.03	AT4G03210.1	*XTH9*

**Table 2:** Differentially expressed genes involved in monolignol and lignan biosynthesis in 21 dai galls versus uninfected roots.

Poplar ID	log_2_ FC	*Arabidopsis* ID	*Arabidopsis* Gene Name
Monolignol Biosynthesis			
Potri.016G091100	1.12	AT2G37040.1	*PAL1*
Potri.006G126800	1.26	AT2G37040.1	*PAL1*
Potri.018G146100	3.47	AT2G30490.1	*ATC4H*, *C4H*, *CYP73A5*, *REF3*
Potri.018G105500	−1.45	AT5G48930.1	*HCT*
Potri.018G105400	−1.22	AT5G48930.1	*HCT*
Potri.001G045500	2.37	AT1G15950.1	*ATCCR1*, *CCR1*, *IRX4*
Potri.008G136600	1.06	AT1G67980.1	*CCOAMT*
Potri.009G099800	1.13	AT4G34050.1	*CCoAOMT1*
Potri.006G169700	−1.21	AT3G21240.1	*4CL2*, *AT4CL2*
Potri.003G188500	−1.21	AT3G21240.1	*4CL2*, *AT4CL2*
Potri.009G063400	−1.16	AT4G37980.1	*ATCAD7*, *CAD7*, *ELI3*, *ELI3-1*
Potri.009G095800	−1.75	AT3G19450.1	*ATCAD4*, *CAD*, *CAD-C*, *CAD4*
Potri.016G078300	1.08	AT4G37970.1	*ATCAD6*, *CAD6*
Potri.002G072100	2.93	AT1G77120.1	*ALCOHOL DEHYDROGENASE 1*
Potri.007G016400	−2.15	AT4G36220.1	*CYP84A1*, *FAH1*
Potri.005G117500	−3.70	AT4G36220.1	*CYP84A1*, *FAH1*
Lignan biosynthesis			
Potri.002G034400	1.26	AT1G75280.1	*PCBER1*
Potri.001G133200	1.79	AT1G32100.1	*ATPRR1*, *PRR1*
Potri.003G100200	1.04	AT1G32100.1	*ATPRR1*, *PRR1*
Potri.001G133300	−2.33	AT1G32100.1	*ATPRR1*, *PRR1*
Methyl donors			
Potri.013G004100	1.56	AT3G17390.1	*MAT4*, *MTO3*, *SAMS3*
Potri.007G147300	1.00	AT2G44160.1	*MTHFR2*

**Table 3:** Differentially expressed genes involved in vascular identity in 21 dai galls versus uninfected roots.

Poplar ID	log_2_ FC	*Arabidopsis* ID	*Arabidopsis* Gene Name
Potri.006G237500	1.01	AT4G32880.1	*ATHB-8*, *ATHB8*, *HB*-8
Potri.010G089200	1.53	AT5G19530.1	*ACL5*
Potri.008G151800	1.60	AT5G19530.1	*ACL5*
Potri.010G217700	1.42	AT1G07900.1	*LBD1*
Potri.012G042100	1.08	AT5G16560.1	*KAN*, *KAN1*
Potri.017G137600	1.16	AT5G16560.1	*KAN*, *KAN1*
Potri.003G096300	1.78	AT1G32240.1	*KAN2*
Potri.006G037000	1.31	AT1G73590.1	*ATPIN1*, *PIN1*
Potri.016G035300	1.59	AT1G73590.1	*ATPIN1*, *PIN1*
Potri.012G019400	1.81	AT3G24770.1	*CLE41*, *TDIF*
Potri.001G049700	2.24	AT4G13195.1	*CLE44*, *TDIF*
Potri.014G104800	1.21	AT2G46770.1	*ANAC043*, *EMB2301*, *NST1*
Potri.011G058400	1.49	AT4G28500.1	*ANAC073*, *NAC073*, *SND2*
Potri.007G135300	1.31	AT4G28500.1	*ANAC073*, *NAC073*, *SND2*
Potri.015G082700	1.06	AT1G57560.1	*AtMYB50*, *MYB50*
Potri.013G113100	2.43	AT1G71930.1	*ANAC030*, *VND7*
Potri.019G083600	2.65	AT1G71930.1	*ANAC030*, *VND7*
Potri.005G129500	−1.13	AT2G23760.1	*BLH4*, *SAW2*
Potri.004G159300	−1.24	AT4G34610.1	*BLH6*
Potri.009G120800	−1.17	AT4G34610.1	*BLH6*
Potri.002G031000	−2.39	AT2G16400.1	*BLH7*
Potri.018G114100	−3.30	AT5G25220.2	*KNAT3*
Potri.006G259400	−1.13	AT5G25220.1	*KNAT3*
Potri.018G095900	−1.63	AT5G57620.1	*AtMYB36*, *MYB36*
Potri.017G017600	−2.63	AT1G17950.1	*ATMYB52*, *BW52*, *MYB52*
Potri.005G064100	−1.70	AT5G64530.1	*ANAC104*, *XND1*
Potri.007G105000	−1.60	AT5G64530.1	*ANAC104*, *XND1*
Potri.001G061200	−1.76	AT5G13180.1	*ANAC083*, *NAC083*, *VNI2*
Potri.013G109300	−1.91	AT1G22640.1	*ATMYB3*, *MYB3*
Potri.017G017600	−2.63	AT1G17950.1	*ATMYB52*, *BW52*, *MYB52*
Potri.002G073500	−2.11	AT1G17950.1	*ATMYB52*, *BW52*, *MYB52*
Potri.005G186400	−1.27	AT1G17950.1	*ATMYB52*, *BW52*, *MYB52*

**Table 4:** Differentially expressed genes involved in phytohormones biosynthesis, signalling and transport in 21 dai galls versus uninfected roots.

Poplar ID	log_2_ FC	*Arabidopsis* ID	*Arabidopsis* Gene Name
Nuclear division and cytokinesis			
Potri.T058000	1.20	AT1G50240.2	*FU*
Potri.019G052500	1.22	AT1G50240.2	*FU*
Potri.004G131600	2.33	AT5G46280.1	*MCM3*
Potri.009G121500	2.34	AT2G16440.1	*MCM4*
Potri.001G070500	2.48	AT1G44900.1	*ATMCM2*, *MCM2*
Potri.018G112800	2.52	AT2G07690.1	*MCM5*
Potri.014G121000	2.23	AT4G02060.1	*MCM7*, *PRL*
Potri.001G074000	2.47	AT5G44635.1	*MCM6*
Potri.006G188700	2.48	AT2G07690.1	*MCM5*
Potri.009G134500	2.55	AT2G20980.1	*MCM10*
Potri.006G131900	1.69	AT3G09660.1	*MCM8*
Cytokinin signalling			
Potri.002G152900	1.32	AT4G16110.1	*ARR2*, *RR2*
Potri.006G041100	1.00	AT3G57040.1	*ARR9*, *ATRR4*
Potri.002G082200	1.27	AT3G57040.1	*ARR9*, *ATRR4*
Potri.018G111300	−1.40	AT2G25180.1	*ARR12*, *RR12*
Auxin biosynthesis and transport			
Potri.018G036800	−1.73	AT5G25620.1	*YUC6*
Potri.002G207400	−1.29	AT1G48910.1	*YUC10*
Potri.004G124200	1.46	AT2G01420.2	*ATPIN4*, *PIN4*
Potri.005G187500	1.57	AT1G77110.1	*PIN6*
Potri.008G129400	1.70	AT1G70940.1	*ATPIN3*, *PIN3*
Potri.002G072200	3.20	AT1G77110.1	*PIN6*
Gibberellins biosynthesis and signalling			
Potri.012G132400	−4.20	AT5G51810.1	*ATGA20OX2*, *GA20OX2*
Potri.015G134600	−1.80	AT5G51810.1	*ATGA20OX2*, *GA20OX2*
Potri.006G247700	−2.82	AT1G15550.1	*ATGA3OX1*, *GA3OX1*, *GA4*
Potri.003G057400	−2.28	AT1G15550.1	*ATGA3OX1*, *GA3OX1*, *GA4*
Potri.010G149700	−1.95	AT1G47990.1	*ATGA2OX4*, *GA2OX4*
Potri.017G124200	3.35	AT1G74670.1	*GASA6*
Potri.012G076700	1.45	AT5G14920.1	*GASA14*
Potri.015G071500	2.70	AT5G14920.1	*GASA14*
Potri.017G021400	−5.17	AT2G01570.1	*RGA*, *RGA1*
Potri.016G027800	−3.35	AT1G14920.1	*GAI*, *RGA2*
Potri.007G133000	−2.48	AT2G01570.1	*RGA*, *RGA1*
Potri.017G125200	−1.17	AT2G01570.1	*RGA*, *RGA1*
Salicylic acid biosynthesis and signalling			
Potri.012G070000	1.16	AT1G74710.1	*ATICS1*, *EDS16*, *ICS1*, *SID2*
Potri.001G288600	1.89	AT2G14610.1	*ATPR1*, *PR* 1, *PR1*
Potri.009G082800	3.42	AT2G14610.1	*ATPR1*, *PR* 1, *PR1*
Potri.009G082900	3.87	AT2G14610.1	*ATPR1*, *PR* 1, *PR1*
Potri.001G288400	4.84	AT2G14610.1	*ATPR1*, *PR* 1, *PR1*
Potri.002G168700	1.98	AT4G23810.1	*ATWRKY53*, *WRKY53*

**Table 5:** List of putatively structurally characterised metabolites with a different abundance in 21 dai galls versus uninfected roots.

Accepted ID	RT (min)	*m*/*z*	*P*-Value	FC
Compounds with increased abundance in 21 dai gall as compared to root				
ferulic acid + sulfate	4.87	273.0063	4.62 × 10^−5^	59.236
tremulacin	20.38	527.1556	0.018	24.870
L-tryptophan	3.33	203.082	0.013	23.719
salicyloyl salicin 1	19.35	405.1186	4.40 × 10^−5^	18.610
G(8-O-4)G(red8-5)G 1	13.33	555.2231	1.75 × 10^−4^	17.631
G(8-O-4)G(red8-5)G 2	13.78	555.2232	1.97 × 10^−5^	10.657
G(e8-O-4)S(8-5)G 2	16.66	583.2178	0.014	8.882
salicortin	8.61	423.1283	1.75 × 10^−5^	6.730
feruloyl hexose 2	5.37	355.103	2.08 × 10^−5^	4.384
G(e8-O-4)S(8-5)G 3	15.80	583.2173	0.003	3.523
salireposide	11.34	405.1186	0.001	2.312
caffeic acid 3/4-O-hexoside 3	4.04	341.0875	0.025	2.158
Compounds with decreased abundance in 21 dai gall as compared to root				
G(t8-O-4)S(8-8)G 1	8.87	583.218	0.033	0.535
quercetin glucoside	8.80	463.0878	1.18 × 10^−4^	0.399
populoside B1	13.03	431.1362	0.011	0.121
caffeic acid 3/4-O-hexoside	2.87	341.0925	0.015	0.074
*p*-coumaroyl quinate 1	4.10	337.0913	0.001	0.053
*p*-coumaroyl hexose 1	3.58	325.0922	8.03 × 10^−6^	0.048
*p*-coumaroyl hexose 3	4.35	325.0909	4.20 × 10^−4^	0.022
chlorogenic acid 2	2.98	353.087	2.83 × 10^−6^	0.007
feruloyl hexose 1	3.03	355.1063	1.36 × 10^−4^	0.004

RT, retention time; FC, fold change.

## Supplementary Materials

Supplementary materials can be found at https://www.mdpi.com/1422-0067/21/2/406/s1. Table S1. Mapping statistics of RNA-Seq experiment; Table S2. Complete list of differentially expressed genes in 21 dai galls versus poplar uninfected root (*p*-value <0.05, log2 ratio>1); Table S3. Complete list of GO families of differentially expressed genes (21 dai galls and uninfected root) as determined with ClueGO and CluePEDIA in Cytoscape; Table S4. Complete metabolomic analysis of 21 dai galls versus uninfected poplar roots; Table S5. Validation of the RNA-seq results by RT-qPCR. Up- and down-regulation are shown in yellow and blue, respectively. FC, fold change.
